# Incidence and Lethality of Suicidal Overdoses by Drug Class

**DOI:** 10.1001/jamanetworkopen.2020.0607

**Published:** 2020-03-23

**Authors:** Ted R. Miller, David I. Swedler, Bruce A. Lawrence, Bina Ali, Ian R. H. Rockett, Nancy N. Carlson, Jennifer Leonardo

**Affiliations:** 1 Pacific Institute for Research and Evaluation, Calverton, Maryland; 2 Curtin University School of Public Health, Perth, Australia; 3 School of Public Health, West Virginia University, Morgantown, West Virginia; 4 American Counseling Association, Alexandria, Virginia; 5 Education Development Center, Waltham, Massachusetts

## Abstract

**Importance:**

Prior lethality analyses of suicide means have historically treated drug poisoning other than alcohol poisoning as a lumped category. Assessing risk by drug class permits better assessment of prevention opportunities.

**Objective:**

To investigate the epidemiology of drug poisoning suicides.

**Design, Setting, and Participants:**

This cross-sectional study analyzed censuses of live emergency department and inpatient discharges for 11 US states from January 1, 2011, to December 31, 2012, as well as Healthcare Cost and Utilization Project national live discharge samples for January 1 to December 31, 2012, and January 1 to December 31, 2016, and corresponding Multiple Cause of Death census data. Censuses or national samples of all medically identified drug poisonings that were deliberately self-inflicted or of undetermined intent were identified using diagnosis and external cause codes. Data were analyzed from June 2019 to January 2020.

**Main Outcomes and Measures:**

Distribution of drug classes involved in suicidal overdoses. Logistic regressions on the state data were used to calculate the odds and relative risk (RR) of death for a suicide act that involved a drug class vs similar acts excluding that class.

**Results:**

Among 421 466 drug poisoning suicidal acts resulting in 21 594 deaths, 19.6% to 22.5% of the suicidal drug overdoses involved benzodiazepines, and 15.4% to 17.3% involved opioids (46.2% men, 53.8% women, and <0.01% missing; mean age, 36.4 years). Opioids were most commonly identified in fatal suicide poisonings (33.3%-47.8%). The greatest RR for poisoning suicide completion was opioids (5.20 times the mean for suicide acts that did not involve opioids; 95% CI, 4.86-5.57; sensitivity analysis range, 3.99-6.86), followed by barbiturates (RR, 4.29; 95% CI, 3.35-5.45), antidepressants (RR, 3.22; 95% CI, 2.95-3.52), antidiabetics (RR, 2.57; 95% CI, 1.94-3.41), and alcohol (conservatively, because 30% of death certifiers do not test for alcohol; RR, 2.04; 95% CI, 1.84-2.26). The updated toxin diagnosis coding in *International Statistical Classification of Diseases and Related Health Problems, Tenth Revision,* used to code the 2016 data revealed that calcium channel blockers also had a high RR of 2.24 (95% CI, 1.89-2.61). Translated to attributable fractions, approximately 81% of suicides involving opioids would not have been fatal absent opioids. Similarly, 34% of alcohol-involved suicide deaths were alcohol attributable.

**Conclusions and Relevance:**

These findings suggest that preventing access to lethal means for patients at risk for suicide should extend to drugs with high case fatality rates. Blister packing and securely storing lethal drugs seems advisable.

## Key Points

**Question:**

What is the association between classes of drugs consumed and the lethality of suicidal drug overdoses?

**Findings:**

In this cross-sectional study of state censuses and national samples that included 421 466 medically identified suicidal drug overdoses, the risk that an overdose would be fatal was highest if an opioid or barbiturate was involved. Lethality increased with age, whereas youth overdoses often involved toxins with low lethality.

**Meaning:**

These findings suggest that drugs that are lethal in overdose when combined should be stored securely in homes, and lethal drugs should be blister packed.

## Teaser

This cross-sectional study investigates the epidemiology of drug poisoning suicides in US state census and national sample data.

## Introduction

Suicide researchers and prevention specialists use the phrase *means matter* to highlight that the mechanism used strongly influences a suicidal act’s lethality.^1^ Through this line of research and practice, means restriction has become a public health focus for population-level suicide prevention.^2^ Analyses of means, however, usually lump almost all drug overdoses into a single category.^2,3,4,5,6,7^ The partial exception is alcohol; a study of fatal and hospital-admitted cases^8^ estimated that people were 5.1 times more likely to attempt suicide when consuming alcohol than when sober. Another small but robust study^9^ estimated that people were 9.6 times more likely to attempt a medically treated suicide during hours when they were intoxicated. Even these studies did not assess how alcohol involvement affected lethality. A few studies on suicide prevention by means focused on restricting access to specific, easily accessible toxins,^10^ such as pesticides, barbiturates, paracetamol (acetaminophen), or antidepressants. Evaluations found that restricting access to these lethal means through prescription practices and sales regulation was associated with declining suicide rates.^10,11,12^ Thus, drug suicide prevention efforts will benefit from a systematic investigation of suicide lethality by drug class that assesses the separate and interactive risks of varied drugs.

This study examined the association of drug classes consumed with the lethality of intentionally self-inflicted drug overdoses. We estimated what proportion of people who died of suicidal acts involving specific drugs may have survived their overdose if their drug mix had omitted a given drug class. For example, we analyzed what portion of completed suicides that involved opioids might not have been fatal if opioids were not involved.

## Methods

This cross-sectional analysis combined mortality and hospital data. Its centerpiece is a tabular and regression analysis of hospital data we previously purchased for unrelated research.^13^ We used mortality data matched by state and year from deidentified, restricted-access Multiple Cause of Death (MCOD) data sets^14^ that recorded the state where the individual died. We restricted analyses to individuals 6 years and older because determining suicidal intent in younger children is often difficult. Most medical examiners do not classify a child’s death as a suicide if the child is younger than 10 years^15^; however, suicide cases have been identified in children aged 5 to 9 years.^16^ This secondary analysis of deidentified data was exempted by the institutional review board of the Pacific Institute for Research and Evaluation, and the need for informed consent was waived. We followed the reporting requirements of the Strengthening the Reporting of Observational Studies in Epidemiology (STROBE) reporting guideline.

We had censuses of deidentified hospital inpatient and emergency department (ED) live discharges from January 1, 2011, through December 31, 2012, from Arizona, Iowa, Nebraska, and Utah for 2012 only; California for 2011 only; and Florida, Kentucky, New Jersey, New York, North Carolina, and Rhode Island for both years. These states accounted for 37% of US residents, 34% of total suicides,^17^ and 39% of drug poisoning suicides in 2012.^18^

To assess the consistency of the 11 states with the nation, we compared their tabular findings vs 2012 Healthcare Cost and Utilization Project (HCUP) National Inpatient Sample (NIS) and Nationwide Emergency Department Sample (NEDS) tables. To ensure that our findings were current, we compared them with 2016 NIS and NEDS tables (the most recent).

Radically different sampling strategies and the absence of state identifiers in HCUP national samples preclude regressions on pooled MCOD, NIS, and NEDS data. The HCUP NIS builds on a 20% sample of discharges from every community hospital in 46 states plus Washington, DC. The HCUP NEDS 2016 sampled discharges from 953 hospitals in 36 states plus Washington, DC; the HCUP NEDS 2012 sampled discharges from 950 hospitals in 30 states. Neither NIS nor NEDS reveals a case’s state of origin. It would be impossible to calculate standard errors (SEs) accurately in a pooled data set regression.

We largely restricted our analysis to deaths and live discharges in which drug poisoning was the mechanism of injury and intent was purposefully self-inflicted or, except in sensitivity analysis, undetermined (hereafter collectively labeled *suicidal*). *International Classification of Diseases (ICD) *intent codes do not differentiate suicidal from nonsuicidal self-inflicted intentional injury. Including drug poisonings of undetermined intent was a conservative correction for documented differential undercounting of drug poisoning suicides across states.^19^ The opioid epidemic is severely stressing underresourced emergency health care and medicolegal death investigation systems across the nation. Drug poisoning suicides are especially difficult for death certifiers to ascertain relative to suicides by more behaviorally and forensically overt methods, such as gunshot and hanging.^20,21^ *Undetermined* is the manner-of-death category most susceptible to obscuring these suicides.^22,23^

We used diagnosis data to determine what drugs, if any, were present in each hospital-treated or fatal poisoning. Presence of alcohol and other drugs was determined using *International Classification of Diseases, Ninth Revision, Clinical Modification* (*ICD-9-CM*) for 2011-2012 hospital data, *International Statistical Classification of Diseases, Tenth Revision, Clinical Modification* (*ICD-10-CM*) for 2016 hospital data, and *International Statistical Classification of Diseases and Related Health Problems, Tenth Revision* (*ICD-10*) for deaths. We identified drug poisonings of suicidal or undetermined intent by any listed diagnosis/intent code in *ICD-10* or external cause code in *ICD-9-CM*. Except in sensitivity analysis, we then excluded cases that also included another suicide mechanism (eg, fall). We classified the drugs involved into the following categories: 4-aminophenol derivative (eg, acetaminophen), alcohol, antiallergic or antiemetic (a single *ICD* code), antidepressant, antipsychotic (ie, tranquilizers other than benzodiazepines), barbiturate, benzodiazepine, cocaine, hallucinogen, opiate, other nonsteroidal anti-inflammatory drug (eg, ibuprofen), muscle relaxant, psychostimulant, salicylate (eg, aspirin), other specified drug, and unknown drug. Because testing only detects components of combination drugs such as acetaminophen and hydrocodone bitartrate, acetaminophen and oxycodone hydrochloride, or cough medicine with codeine, these drugs were counted both as an opioid and as 1 or more other drug classes. The 2016 analysis differentiated additional drug classes identifiable only in *ICD-10*. eTable 1 in the Supplement lists the codes included in each drug class. A maximum of 8 classes were involved in 1 case. Data management and descriptive statistics were conducted with SAS, version 9.4 (SAS Institute Inc) and RStudio, version 1.2.5033 (RStudio Inc).

Drugs involved in suicide acts were not always tested or identifiable. Fewer than 10% of nonfatal poisonings but almost 45% of fatal poisonings included unknown drugs. If the record identified any drugs, we ignored the unknown drugs. For fatalities with all drugs unknown, we used a published regression method^24^ to infer the presence of frequently involved drug classes, that is, opioids, benzodiazepines, and stimulants (with probabilities >0.33 counted as present). We followed a similar strategy for nonfatal cases but could only use victim sex, age group, and rurality as explanators.

We tabulated the data. By time period, we computed the percentage of cases that were fatal (ie, the case fatality rate) and the risk of suicide due to drug poisoning involving each drug class relative to drug poisonings that did not involve that class (ie, the incidence rate ratio or the risk ratio).

### Statistical Analysis

Data were analyzed from June 2019 to January 2020. Our 2011-2012 state fatality and hospital data were censuses suitable for regression analysis after pooling. Using Stata, version 15.0 (StataCorp LLC), we ran a series of logistic regressions on them. The regressions estimated the odds that a poisoning suicidal act was fatal, with fatal cases equal to 1 and nonfatal cases equal to 0.

Covariates included victim age group (detailed in Table 1) by sex, 3-category urbanicity (large metropolitan/fringe, medium/small metropolitan, and micropolitan/noncore), and state. The HCUP data include urbanicity; we generated comparable categories from state and county information in the MCOD. The ED data offered no other demographic characteristics.

**Table 1.** Case Counts of Suicidal Drug Poisoning Overdoses by Characteristics and Year^a^

Characteristic	2011-2012 Events in 11 States			2012 National Events			2016 National Events		
	Deaths (n = 5081)	Admissions (n = 120 623)	ED discharges (n = 120 658)	Deaths (n = 9693)	Weighted admissions (SE) (n = 225 609)	Weighted ED discharges (SE) (n = 207 365)	Deaths (n = 10 525)	Weighted admissions (SE) (n = 155 610)	Weighted ED discharges (SE) (n = 139 160)
Male	2696	50 439	52 452	5190	99 115 (538)	85 095 (501)	5660	59 618 (419)	52 970 (405)
Age, y									
6-14	9	2460	6105	12	10 552 (223)	4765 (153)	26	11 409 (222)	4240 (143)
15-20	114	13 839	25 098	225	45 657 (429)	24 325 (328)	274	39 757 (375)	20 160 (294)
21-25	249	12 000	16 364	486	31 338 (368)	20 470 (304)	571	20 787 (290)	14 885 (258)
26-30	305	11 389	13 480	698	26 261 (341)	19 475 (297)	783	17 046 (266)	13 090 (244)
31-39	668	19 539	19 145	1431	36 508 (393)	38 495 (396)	1687	23 989 (266)	23 225 (311)
40-49	1287	26 345	19 931	2446	36 145 (392)	42 270 (410)	2207	20 582 (290)	24 955 (320)
50-59	1445	21 556	13 615	2746	23 900 (324)	36 440 (387)	2726	15 705 (258)	23 045 (310)
≥60	1004	13 413	6675	1649	11 769 (231)	20 730 (305)	2251	7334 (180)	15 560 (263)
Undetermined intent	1211	27 954	50 234	2952	93 556 (538)	44 055 (417)	3809	31 981 (346)	15 730 (264)
With comorbidities	760	112 017	73 080	NA	NA	NA	NA	NA	NA
Drug classes per case (excluding alcohol), mean (SE)	1.37 (0.01)	1.38 (0.002)	1.04 (0.002)	1.30 (0.01)	1.06 (0.002)	1.41 (0.004)	1.40 (0.01)	1.21 (0.003)	1.45 (0.01)
4-Aminophenol derivative	278	15715	9435	393	16 159 (273)	26 950 (342)	387	14 228 (246)	19 355 (289)
Alcohol	550	8621	7535	1123	11 038 (231)	14 535 (260)	1429	7153 (178)	11 080 (226)
Antiallergic/antiemetic	373	5218	4238	602	8461 (207)	10 580 (224)	813	8610 (195)	9225 (208)
Antidepressant	851	16 567	10 160	1615	20 696 (311)	32 445 (370)	1714	21 519 (297)	24 240 (316)
Antidiabetic	55	2318	845	87	1603 (85)	4400 (147)	117	1277 (78)	3670 (134)
Antiepileptic	282	6802	4580	471	8942 (212)	12 145 (239)	688	9665 (206)	13 960 (251)
Antiparkinsonian	81	2881	2449	140	3933 (136)	4775 (153)	123	2117 (99)	3635 (133)
Barbiturate	95	1146	307	150	555 (50)	1865 (96)	157	268 (35)	865 (66)
Benzodiazepine	808	34 291	20 372	1245	39 026 (414)	58 700 (459)	1551	25 283 (316)	33 020 (355)
Cocaine	130	7010	2520	228	4495 (144)	8200 (198)	470	2014 (95)	3585 (132)
Hallucinogen	13	1917	2426	28	4652 (146)	3075 (123)	28	2478 (106)	1990 (99)
Muscle relaxant	3	2707	1280	7	2646 (113)	5505 (164)	219	2077 (99)	3115 (124)
Opioid	1981	19 749	16 148	3226	35 350 (381)	33 250 (374)	4386	26 196 (319)	21 635 (302)
Other analgesic	5	380	426	7	746 (62)	510 (50)	4	535 (50)	380 (44)
Other antipsychotic	282	10 818	6070	500	11 202 (233)	19 250 (295)	653	11 978 (228)	16 970 (273)
Other NSAID	26	4796	7117	39	13 515 (248)	8680 (204)	36	14 956 (251)	8515 (200)
Psychostimulant	116	3436	2996	218	6138 (171)	7040 (184)	417	5843 (163)	5635 (164)
Salicylate	58	3237	1936	100	3397 (128)	6085 (172)	69	2812 (114)	3830 (136)
Other named drug class	546	22 435	15 955	904	31 055 (366)	40 905 (405)	722	18 357 (276)	21 665 (302)
Unknown drug only	704	3523	15 066	1470	28 001 (353)	9915 (217)	1522	19 115 (279)	6920 (181)

Abbreviations: ED, emergency department; NA, not applicable; NSAID, nonsteroidal anti-inflammatory drug.

^a^ Unweighted counts are given in eTable 1 in the Supplement. Unless otherwise indicated, data are expressed as number of cases.

Regressions included the presence of comorbidities as a covariate. We searched the record for the 30 Elixhauser comorbid conditions.^25^ Hospitals assiduously code comorbidities, which can affect treatment choices or require maintaining a medication regimen or diet. Conversely, death certifiers only record comorbidities that contributed to the death.^26^ Therefore, we treated comorbidity as an absent-present variable rather than a count.

Three overarching regression models covered all drug classes. Model 1 included binary variables indicating the drug classes involved. Models 2 and 3 were sensitivity analyses that paralleled model 1. Model 2 omitted cases in which none of the drug classes involved were known or inferable. Model 3 omitted cases of undetermined intent. Because exploratory data tabulations suggested youths used different drug mixes than adults, we also ran model 1 separately for those 21 years or older and younger than 21 years.

Additional regressions further probed the lethality of the 9 classes of drugs with at least 100 deaths in the 2011-2012 sample. These regressions (model 4) added a set of interaction terms between the binary indicator variable for the drug class of interest and the indicator variables for the other drug classes. We calculated the increased lethality if a focal drug class was involved in a suicide act in 2 steps.^27^ First, we summed the regression coefficients for the drug class indicator and its interaction terms multiplied by the percentage of a drug class’ cases that involved each interaction. Then we exponentiated the sum to generate the adjusted odds ratio. Model 4 provides our best estimates. Model 1 is best for drug classes with too few deaths to calculate model 4 reliably. By including cases with only unknown drugs and cases of undetermined intent, these models should reduce the biases resulting from differential coding in the fatal and nonfatal data.

Model 5 paralleled model 1 but included suicidal acts from all mechanisms, with binary variables indicating the primary mechanism. The findings appear in eTable 2 in the Supplement.

We converted the odds ratios to relative risks (RRs) using a correction formula that derives directly from the definitions of the 2 terms.^28^ That formula is apropos because we have a census of cases. For RRs exceeding 1.00, we computed the fraction of deaths involving the focal drug class that would not have been fatal if no drugs in that class had been present. We calculated this drug-attributable fraction among the exposed by subtracting the base risk of 1.00 from the RR and then dividing by the RR.^29,30^

## Results

A total of 421 466 drug poisoning suicidal acts resulted in 21 594 deaths (46.2% men, 53.8% women, and <0.01% missing; mean age, 36.4 years). Table 1 and Table 2 as well as eTable 3 in the Supplement describe the suicidal acts and drug classes analyzed in the 3 populations: 246 362 events in the 2011-2012 state censuses (including 5081 deaths), 102 946 events (unweighted) in the 2012 national data (including 9693 deaths, with 48 340 nonfatal events and 1211 deaths also in the state censuses), and 73 369 events in the 2016 national data (including 10 525 deaths). In 2016, a nonfatal act treated in the ED involved a mean (SE) of 1.45 (0.01) drug classes; an admitted act, 1.21 (0.003) drug classes; and a fatality, 1.40 (0.004) drug classes, excluding alcohol. Across the 3 measurements, benzodiazepines were involved in 19.6% to 22.5% of the suicidal overdoses (Table 2). They were the drug class most often involved in nonfatal acts. Among fatalities, opioids (33.3%-47.8% of cases), antidepressants (16.3%-16.7%), and benzodiazepines (12.8%-15.9%) were most often involved.

**Table 2.** Distribution of Suicidal Acts Between Categories, Distribution of Drugs Involved in Fatal Cases, and Unadjusted RRs of Death by Characteristics and Year

Characteristic	Suicidal acts, %						RR (95% CI)		
	State cases 2011-2012	National cases 2012	National cases 2016	State deaths 2011-2012	National deaths 2012	National deaths 2016	2011-2012	2012	2016
Male	42.9	42.8	38.7	53.1	53.5	53.8	1.51 (1.43-1.59)	1.60 (1.54)	1.94 (1.87-2.02)
Age, y									
6-14	3.5	3.5	5.1	0.2	0.1	0.2	0.05 (0.02-0.08)	0.04 (0.02-0.06)	0.05 (0.03-0.07)
15-20	15.9	15.9	19.7	2.2	2.3	2.6	0.12 (0.10-0.15)	0.13 (0.11-0.15)	0.11 (0.10-0.13)
21-25	11.6	11.8	11.9	4.9	5.0	5.4	0.39 (0.35-0.44)	0.40 (0.37-0.44)	0.44 (0.41-0.48)
26-30	10.2	10.5	10.1	6.0	7.2	7.4	0.56 (0.50-0.63)	0.69 (0.63-0.73)	0.74 (0.69-0.79)
31-39	16.0	17.3	16.0	13.1	14.8	16.0	0.80 (0.73-0.86)	0.86 (0.81-0.90)	1.04 (0.99-1.09)
40-49	19.3	18.3	15.6	25.3	25.2	21.0	1.42 (1.33-1.51)	1.55 (1.48-1.62)	1.49 (1.42-1.56)
50-59	14.9	14.3	13.6	28.4	28.3	25.9	2.28 (2.14-2.42)	2.44 (2.33-2.55)	2.31 (2.21-2.41)
≥60	8.6	7.7	8.2	19.8	17.0	21.4	2.63 (2/46-2.81)	2.51 (2.38-2.65)	3.14 (3.01-3.29)
Undetermined intent	32.2	31.8	16.9	32.2	30.5	36.2	0.66 (0.62-0.70)	0.97 (0.93-1.01)	2.91 (2.80-3.02)
With comorbidities	85.0	NA	NA	15.0	NA	NA	0.06 (0.05-0.06)	NA	NA
4-Aminophenol derivative	11.2	9.9	11.1	5.5	4.1	3.7	0.50 (0.44-0.57)	0.40 (0.36-0.44)	0.32 (0.28-0.35)
Alcohol	6.8	6.1	6.4	10.8	11.6	13.6	1.67 (1.53-1.82)	2.09 (1.97-2.22)	2.37 (2.24-2.50)
Antiallergic/antiemetic	4.0	4.4	6.1	7.3	6.2	7.7	1.91 (1.71-2.11)	1.46 (1.34-1.58)	1.33 (1.24-1.43)
Antidepressant	11.2	12.4	15.5	16.7	16.7	16.3	1.60 (1.49-1.71)	1.45 (1.38-1.53)	1.10 (1.05-1.16)
Antidiabetic	1.3	1.4	1.7	1.1	0.9	1.1	0.83 (0.62-1.05)	0.66 (0.53-0.81)	0.69 (0.56-0.82)
Antiepileptic	4.7	4.9	8.0	5.6	4.9	6.5	1.18 (1.04-1.33)	1.02 (0.92-1.11)	0.84 (0.78-0.90)
Antiparkinsonian	2.2	2.0	1.9	1.6	1.4	1.2	0.72 (0.57-0.88)	0.73 (0.62-0.86)	0.62 (0.52-0.73)
Barbiturate	0.6	0.6	0.4	1.9	1.5	1.5	3.01 (2.43-3.60)	2.75 (2.34-3.19)	3.69 (3.14-4.25)
Benzodiazepine	22.5	21.4	19.6	15.9	12.8	14.7	0.65 (0.60-0.70)	0.53 (0.50-0.56)	0.74 (0.70-0.78)
Cocaine	3.9	2.9	2.0	2.6	2.4	4.5	0.64 (0.53-0.76)	0.82 (0.72-0.93)	2.39 (2.18-2.60)
Hallucinogen	1.8	1.8	1.5	0.3	0.3	0.3	0.14 (0.08-0.23)	0.17 (0.11-0.23)	0.18 (0.12-0.26)
Muscle relaxant	1.6	1.8	1.8	0.1	0.1	2.1	0.04 (0.00-0.08)	0.04 (0.01-0.07)	1.22 (1.06-1.38)
Opioid	15.4	16.1	17.3	39.0	33.3	47.8	3.52 (3.32-3.71)	2.64 (2.53-2.75)	4.55 (4.38-4.73)
Other analgesic	0.3	0.3	0.3	0.1	0.1	0.0	0.30 (0.06-0.60)	0.26 (0.07-0.48)	0.13 (0.03-0.26)
Other antipsychotic	7.0	7.0	9.7	5.6	5.2	6.2	0.78 0.69-0.88)	0.74 (0.68-0.81)	0.64 (0.59-0.69)
Other NSAID	4.8	5.0	7.7	0.5	0.4	0.3	0.10 (0.06-0.14)	0.08 (0.05-0.10)	0.04 (0.03-0.06)
Psychostimulant	2.7	3.0	3.9	2.3	2.2	4.0	0.86 (0.70-1.01)	0.75 (0.65-0.86)	1.05 (0.95-1.16)
Salicylate	2.1	2.2	2.2	1.1	1.0	0.7	0.53 (0.40-0.67)	0.48 (0.39-0.60)	0.30 (0.23-0.38)
Other named drug class	15.8	16.0	13.4	10.7	9.3	7.5	0.64 (0.59-0.70)	0.54 (0.50-0.57)	0.54 (0.51-0.58)
Unknown drug only	7.8	8.9	7.9	3.6	15.2	14.5	1.89 (1.74-2.04)	1.87 (1.77-1.98)	1.77 (1.67-1.86)

Abbreviations: NA, not applicable; NSAID, nonsteroidal anti-inflammatory drug; RR, relative risk.

The case fatality rate (eTable 3 in the Supplement) measures lethality. It was higher for males (2.6%-4.8%) than females (1.7%-2.6%) and increased with age group (6-14 years, 0.1%-0.2%; ≥60 years, 4.8%-9.0%). It consistently was highest for opioids (4.5%-9.5%), followed by antidepressants (2.9%-3.6%) and barbiturates (5.8%-12.2%). eTable 4 in the Supplement shows the percentage of cases within a given drug class for which that drug class was the only class identified for fatal and nonfatal cases. Among fatalities, antidiabetics (81.8%) and salicylates (60.3%) were the 2 drug classes that often were taken alone.

Opioids (RR, 5.20; 95% CI, 4.86-5.57) and barbiturates (RR, 4.29; 95% CI, 3.35-5.45) consistently had the highest unadjusted RRs in all periods. That pattern persisted in the regression-adjusted RRs shown in Table 3. The risk of a suicide act resulting in death was highest if opioids were involved, with a best estimate that death was 5.18 (95% CI, 4.81-5.58) times more likely. Across sensitivity analyses, this estimate ranged from 3.99 to 6.86. As Table 3 shows, this estimate translates as 75% to 87% of the suicide deaths involving opioids would not have been fatal if opioids were not involved.

**Table 3.** Relative Risks and Attributable Fractions Derived From Logistic Regressions

Variable	RR (95% CI)		Sensitivity analysis range, estimate from models 2, 3, and 5^b^
	Linear model 1	Cross-product model 4^a^	
4-Aminophenol derivative	0.94 (0.82 to 1.09)	0.86 (0.71 to 1.03)	0.63 to 1.06
Alcohol	2.04 (1.84 to 2.26)	1.50 (1.32 to 1.72)	2.00 to 2.49
Antiallergic/antiemetic	3.94 (3.48 to 4.45)	1.17 (0.98 to 1.39)	2.69 to 4.72
Antidepressant	3.22 (2.95 to 3.52)	2.75 (2.48 to 30.5)	1.95 to 3.82
Antidiabetic	2.57 (1.94 to 3.41)	NA	2.13 to 3.06
Antiepileptic	1.20 (1.05 to 1.39)	0.91 (0.75 to 1.10)	0.83 to 1.43
Antiparkinsonian	0.67 (0.53 to 0.87)	NA	0.64 to 0.78
Barbiturate	4.29 (3.35 to 5.45)	NA	4.26 to 4.64
Benzodiazepine	0.71 (0.66 to 0.78)	0.44 (0.39 to 0.49)	0.43 to 0.85
Cocaine	1.30 (1.08 to 1.58)	1.10 (0.85 to 1.40)	1.09 to 1.64
Hallucinogen	0.20 (0.11 to 0.35)	NA	0.18 to 0.23
Muscle relaxant	0.04 (0.01 to 0.14)	NA	0.05 to 0.05
Opioid	5.20 (4.86 to 5.57)	5.18 (4.81 to 5.58)	3.99 to 6.86
Other analgesic	0.47 (0.18 to 1.16)	NA	0.42 to 0.60
Other antipsychotic	1.50 (1.31 to 1.73)	1.24 (0.85 to 1.40)	0.99 to 1.74
Other NSAID	0.24 (0.16 to 0.35)	NA	0.16 to 0.26
Psychostimulant	1.44 (1.17 to 1.77)	1.26 (0.98 to 1.63)	1.22 to 1.72
Salicylate	1.59 (1.20 to 2.10)	NA	1.16 to 1.85
Other named drug class	1.18 (1.07 to 3.18)	NA	1.05 to 1.42
Unknown drug only	2.92 (2.64 to 3.20)	NA	1.90 to 4.28
Attributable fraction^c^			
4-Aminophenol derivative	NA	NA	0.06 to 2.72
Alcohol	0.51 (0.46 to 0.56)	0.34 (0.24 to 0.42)	0.50 to 0.61
Antiallergic/antiemetic	0.75 (0.71 to 0.78)	0.14 (−0.02 to 0.28)	0.63 to 0.80
Antidepressant	0.69 (0.66 to 0.72)	0.64 (0.60 to 0.67)	0.49 to 0.75
Antidiabetic	0.61 (0.48 to 0.71)	NA	0.53 to 0.69
Antiepileptic	0.17 (0.05 to 0.28)	−0.10 (−0.33 to 0.09)	NA
Antiparkinsonism	NA	NA	NA
Barbiturate	0.77 (0.70 to 0.82)	NA	0.77 to 0.80
Benzodiazepine	NA	NA	NA
Cocaine	0.23 (0.07 to 0.37)	0.09 (−0.17 to 0.28)	0.08 to 0.40
Hallucinogen	NA	NA	NA
Muscle relaxant	NA	NA	NA
Opioid	0.81 (0.79 to 0.82)	0.81 (0.79 to 0.82)	0.75 to 0.87
Other analgesic	NA	NA	NA
Other antipsychotic	0.33 (0.24 to 0.42)	0.20 (0.05 to 0.32)	0.44 to 101.0
Other NSAID	NA	NA	NA
Psychostimulant	0.30 (0.14 to 0.44)	0.21 (−0.02 to 0.39)	0.18 to 0.43
Salicylate	0.37 (0.16 to 0.52)	NA	0.13 to 0.47
Other named drug class	0.15 (0.06 to 0.69)	NA	0.05 to 0.30
Unknown drug only	0.66 (0.62 to 0.69)	NA	0.47 to 0.78

Abbreviations: NA, not applicable; NSAID, nonsteroidal anti-inflammatory drug; RR, relative risk.

^a^ A cross-product model was not run if fewer than 100 fatalities were in the drug class. No cross-product model was run on unknown drugs.

^b^ Odds ratios and associated 95% CIs underlying the RRs in the sensitivity analyses appear in eTable 2 and eTable 5 in the Supplement.

^c^ Calculated if the RR exceeded 1.00.

Relative to the fatality risk for all other drugs combined, antiallergics or antiemetics (RR, 3.94; 95% CI, 3.48-4.45), antidepressants (RR, 3.22; 95% CI, 2.95-3.52), and alcohol (RR, 2.04; 95% CI, 1.84-2.26) had high RRs in the linear model (model 1), but their association was attenuated in a cross-product model (model 4) that accounted for the presence of other drugs. Too few cases involved barbiturates to test whether its high RR would attenuate. The RR for antidiabetics almost certainly would not attenuate because more than 80% of those deaths involved no other drugs. Besides opioids (81%), the attributable fraction of deaths involving another drug only exceeded 0.5 for barbiturates (77%), antidepressants (64%), and antidiabetics (61%). Among alcohol-involved deaths, 34% were alcohol attributable.

eTable 2 and eTable 5 in the Supplement provide the full regression models that produced the results in the sensitivity analysis column in Table 3. eTable 6 in the Supplement displays the regressions underlying the interaction models.

Table 4 as well as eTable 7 and eTable 8 in the Supplement compare drug suicide patterns of youths and adults. After controlling for drugs chosen, lethality was lower for youths than adults. The drug mixes chosen by youths and adults contrasted markedly. Youth poisoning suicide acts were more likely to include nonopioid pain relievers (eg, acetaminophen, aspirin, or ibuprofen), antidepressants, and antiallergic or antiemetic drugs. In contrast, attempts in adults were more likely to include alcohol, benzodiazepines, cocaine, opioids, and drugs to treat chronic conditions (eg, epilepsy, Parkinson disease). The adult regression yielded almost identical odds ratios by drug to the all-age model. The youth regression was hampered by sample size. With a broad 95% CI, the RRs of death for opioids (8.36 [95% CI, 5.36-13.00] vs 5.07 [95% CI, 4.73-5.42]) and barbiturates (9.43 [95% CI, 1.26-62.26] vs 4.22 [95% CI, 3.31-5.36]) were larger for youths than adults. Conversely, alcohol involvement only was associated with increased risk among adults (RR, 2.06 [95% CI, 1.86-2.29] vs 0.25 [95% CI, 0.03-1.85]).

**Table 4.** Youth and Adult Toxin Distributions, RRs, and Attributable Fractions for 2011-2012 State Data

Drug	Distribution, No. (%)						RR (95% CI)			
	Youth cases (n = 47 625)	Adult cases (n = 198 737)	Youth deaths (n = 123)	Adult deaths (n = 4958)	Youth admissions (n = 16 299)	Adults admissions (n = 104 324)	Youths	Adults	Adjusted youths	Adjusted adults
4-Aminophenol derivative	7658 (1.6)	54 834 (17.4)	6 (4.9)	272 (5.5)	3182 (23.4)	11 893 (11.4)	0.27 (0.08-0.51)	0.59 (0.52-0.66)	0.43 (0.19-1.01)	0.97 (0.84-1.12)
Alcohol	1723 (3.6)	17 751 (8.9)	1 (0.8)	549 (11.1)	576 (3.5)	8044 (7.7)	0.22 (0.00-0.73)	1.52 (1.39-1.66)	0.25 (0.03-1.85)	2.06 (1.86-2.29)
Antiallergic/antiemetic	2906 (6.1)	6911 (3.5)	11 (8.9)	362 (7.3)	1246 (7.6)	3968 (3.8)	1.51 (0.71-2.55)	2.18 (1.96-2.41)	2.13 (1.07-4.27)	3.98 (3.51-4.51)
Antidepressant	5841 (12.3)	21 730 (10.9)	17 (13.8)	834 (16.8)	2670 (16.4)	13 893 (13.3)	1.15 (0.61-1.83)	1.64 (1.53-1.76)	2.43 (1.38-4.27)	3.21 (2.94-3.51)
Antidiabetic	361 (0.8)	2856 (1.4)	1 (0.8)	54 (1.1)	218 (1.3)	2100 (2.0)	1.07 (0.00-3.60)	0.75 (0.56-0.95)	2.78 (0.37-19.95)	2.55 (1.92-3.37)
Antiepileptic	1156 (2.4)	10 507 (5.3)	2 (1.6)	280 (5.6)	473 (2.9)	6329 (6.1)	0.66 (0.00-1.73)	1.07 (0.95-1.20)	0.77 (0.19-3.14)	1.21 (1.05-1.40)
Antiparkinsonian	419 (0.9)	4987 (2.5)	1 (0.8)	80 (1.6)	173 (1.1)	2705 (2.6)	0.92 (0.00-3.13)	0.64 (0.50-0.78)	0.61 (0.08-4.47)	0.69 (0.54-0.87)
Barbiturate	101 (0.2)	1447 (0.7)	1 (0.8)	94 (1.9)	65 (0.4)	1081 (1.0)	3.86 (0.00-13.07)	2.63 (2.12-3.16)	9.43 (1.26-62.26)	4.22 (3.31-5.36)
Benzodiazepine	4565 (9.6)	50 896 (25.6)	10 (8.8)	798 (16.1)	1951 (12.0)	32 336 (31.0)	0.83 (0.34-1.45)	0.56 (0.52-0.60)	0.71 (0.36-1.40)	0.72 (0.66-0.78)
Cocaine	381 (0.7)	9320 (4.7)	0	130 (2.6)	163 (1.0)	6833 (6.5)	NA	0.55 (0.45-0.64)	NA	1.33 (1.10-1.60)
Hallucinogen	1489 (3.1)	2863 (1.4)	1 (0.8)	12 (0.2)	390 (2.4)	1525 (1.5)	0.25 (0.00-0.87)	0.55 (0.45-0.64)	0.18 (0.03-1.33)	0.20 (0.11-0.37)
Muscle relaxant	434 (0.9)	3555 (1.8)	0	3 (0.1)	227 (1.4)	2480 (2.4)	NA	0.03 (0.00-0.08)	NA	0.04 (0.01-0.14)
Opioid	3318 (7.0)	34 548 (17.4)	45 (36.6)	1936 (39.0)	1248 (7.7)	18 496 (17.7)	7.70 (5.23-10.95)	3.04 (2.88-3.21)	8.36 (5.36-13.00)	5.07 (4.73-5.42)
Other analgesic	3203 (0.9)	13 957 (7.0)	6 (4.9)	276 (5.6)	1522 (9.3)	9294 (8.9)	NA	0.36 (0.07-0.71)	NA	0.49 (0.19-1.21)
Other antipsychotic	1823 (7.0)	4712 (2.4)	6 (4.9)	110 (2.2)	669 (4.1)	2763 (2.6)	0.71 (0.22-1.39)	0.78 (0.69-0.88)	1.11 (0.47-2.61)	1.51 (1.31-1.74)
Other NSAID	5593 (0.5)	6340 (3.2)	0	26 (0.5)	1791 (11.0)	3003 (2.9)	NA	0.16 (0.10-0.22)	NA	0.27 (0.18-0.40)
Psychostimulant	1723 (6.7)	14 979 (7.5)	1 (0.8)	549 (11.1)	576 (3.5)	8044 (7.7)	1.29 (0.38-2.49)	0.93 (0.76-1.11)	1.31 (0.55-3.09)	1.43 (1.16-1.77)
Salicylate	2040 (3.8)	3189 (1.6)	4 (3.3)	54 (1.1)	1031 (6.3)	2204 (2.1)	0.75 (0.17-1.61)	0.67 (0.50-0.86)	1.61 (0.57-4.47)	1.58 (1.17-2.10)
Other named drug class	8246 (17.3)	30 642 (15.4)	17 (13.8)	529 (10.7)	3471 (21.3)	18 950 (18.2)	0.77 (0.42-1.21)	0.65 (0.60-0.71)	1.06 (0.62-1.84)	1.17 (1.06-1.30)
Unknown drug only	4843 (10.2)	14 423 (7.3)	11 (8.9)	693 (14.0)	639 (3.9)	2833 (2.8)	0.87 (0.43-1.47)	2.07 (1.91-2.24)	0.98 (0.48-1.97)	2.98 (2.71-3.28)

Abbreviations: NA, not applicable; NSAID, nonsteroidal anti-inflammatory drug; RR, relative risk.

The 2012 patterns for the 11 states and the nation shown in Table 2 were similar. The 2012 and 2016 national data were similar as well, except opioid-involved fatalities increased from 33.3% to 47.8%. Probing the data revealed that this resulted exclusively from deaths involving fentanyl and other synthetic opioids more than tripling from 516 to 1798 deaths.

A few drugs frequently involved in suicide acts are identifiable in *ICD-10* but not *ICD-9-CM*. Relative risks for those drugs, based on 2016 data unadjusted for demographic characteristics and comorbidities, appear in eTable 9 in the Supplement. The attributable fraction of involved deaths for calcium channel blockers was high at 55% (RR, 2.24; 95% CI, 1.89-2.61).

## Discussion

This analysis found that including opioids or barbiturates in a suicidal drug mix increased the risk that a suicide attempt would prove lethal. The increase in lethality of suicidal opioid overdoses from 2012 to 2016 paralleled the rise in unintentional overdose deaths, which resulted from the spread of nonpharmaceutical fentanyl and analogues.^31^

Adults were much more likely than youths to include highly lethal drugs in their suicidal mix. The descriptive data suggest that youths took whatever was at hand, including nonopioid analgesics, allergy medications, or anything easily accessible.^32^ This finding emphasizes the importance of locking up drugs prescribed to any family member if they can be lethal in overdose.^33^ Parents of children with depression or who have multiple risks probably would be wise to compile a list of all their family’s medications and ask their pharmacist to check for drugs that are likely to be lethal if combined in an overdose. Disposing of leftover drugs may also help prevent a fatal suicide attempt.^34^

Limiting access to lethal means is a proven way to prevent suicides.^35,36,37,38^ Smaller pack size and blister packaging proved protective against suicide by paracetamol.^11,39^ To reduce suicide risk, we recommend blister packing all opioids and other potentially lethal drugs.^40,41^ Suicidal acts often are impulsive, and once underway, the individual is impatient.^42^ Youths, in particular, are likely to simply choose a different bottle rather than patiently empty blister packs.

Adults used more considered drug mixes than youths. Their mixes increased lethality. It is unclear why adults had higher mortality rates than youths when they included alcohol in their mix. Possibly they took more drugs that interacted adversely with alcohol. Conversely, alcohol use disorder is a known risk factor for suicide.^43,44,45^ Therefore, alcohol involvement among adults might serve as a marker for alcohol use disorders that focused attempters on more lethal drug mixes. Antidepressant involvement might provide a similar signal.

The opioid-involved suicide rate more than doubled from 1999 and 2009, then stabilized.^46^ In reporting the death toll from the opioid epidemic, analysts routinely include these opioid-involved suicides.^47,48,49^ Our analysis confirms the wisdom of including them.^47^ It suggests that 75% to 87% of these deaths would not have occurred if the decedent had lacked access to opioids. Suicide acts often are impulsive and use readily accessible, often previously ideated means, so those who intentionally overdosed on opioids would be likely to try a drug overdose even if opioids were not readily available.^35,36^ However, without opioids in the mix, it appears that they probably would survive.

Reducing opioid involvement could save lives beyond the short term. A meta-analysis of predominantly drug poisoning cases found only 2.1% of suicide survivors died by suicide in the following 2 years.^50^ Indeed, means matter.

### Limitations

This study has some limitations. Our analysis was hampered by coding deficiencies in mortality and hospital data. First, only a subset of drug self-intoxication cases coded as undetermined intent were suicidal.^47^ Of the fatalities in Table 1, 27.6% were of undetermined intent, whereas none of the drugs involved were identified in 14.6% of cases. For nonfatal cases in Table 1, these percentages were 27.1% and 8.5%, respectively. Furthermore, 30% of death certifiers would not have tested for alcohol in drug poisonings, so our alcohol-attributable fraction is probably an underestimate.^51^ Our regression-based estimates of the attributable fractions omitting cases with undetermined intent and unknown drug were similar to estimates including them. Second, we unavoidably omitted fatal and nonfatal suicide acts that were deliberately miscoded as unintentional to avoid stigma or insurance problems. Third, we unavoidably omitted suicide acts among the 8.3% of *ICD-9-CM* drug poisoning cases that lacked external cause codes. Although poisoning intent cannot be omitted in *ICD-10-CM*, coders are encouraged to code as unintentional rather than undetermined. The net effect of these omissions and overinclusions is unclear. Nevertheless, some readers may prefer to view the results omitting undetermined intent (model 3) as primary.

Differences in drug coding between *ICD* editions and sparse counts also limited our analysis. The *ICD-9-CM*, which underpinned our regression-adjusted estimates, lumped other drugs with cannabis, β-blockers, and calcium channel blockers. We had too few cases involving most drugs to use cross-product regressions that adjusted for drug interactions. Consequently, we could only provide 2016 unadjusted RR estimates for those drugs. For some other drugs, we saw dramatic declines in RR from unadjusted or linear regression to cross-product regression estimates. Regressions using pooled 2016 and 2017 state census data would improve our estimates.

Our data also lack recency. Comparing 2012 and 2016 data, however, suggested that the risk pattern has been stable for all drugs except opioids.

Our regression results may not be nationally representative. We used 2011-2012 data from a convenience sample of states that made affordable hospital discharge censuses available. Although we lack data from the Appalachian states that were hard hit by the opioid epidemic, we have state data from the coasts and heartland, as well as populous and sparsely populated states. Furthermore, the data sets lacked important demographic explanators, including race, ethnicity, and income. Our concerns about representativeness are softened by the consistency of the RRs in the 2011-2012 data before regression adjustment with the national RRs for 2012.

## Conclusions

The suicide prevention concept of “means matter” extends to drug class within poisoning suicides. This study appears to reemphasize the need to control access to drug classes that increase the risk of dying of a suicidal overdose. This study suggests that lethal drug access is particularly an issue for youths because they rarely take a targeted set of drugs, seemingly opting for whatever is accessible.

## Notes

Supplement. **eTable 1.** Drug Codes for Poisonings of Suicidal and Undetermined Intent, by Coding System **eTable 2.** Regression Model for 2011-2012 Data Including All Suicide Mechanisms (n = 415 595) **eTable 3.** Case Fatality Rates (CFR), Case Counts of Suicidal Intent-Only Drug Poisoning Overdoses, and Unweighted Case Counts for 2012 and 2016 NIS/NEDS Data **eTable 4.** Percent of Cases in the 2011-2012 Overdoses Where a Given Drug Class Was the Only Drug Class Identified **eTable 5.** Complete Regression Output for Model 1 and Sensitivity Analyses for Models Excluding Unknown Drugs and Excluding Cases of Undetermined Intent **eTable 6.** Regression Models and Coefficients for Drug Interactions Used to Generate Combined Odds Ratios **eTable 7.** Drug Frequencies by Setting for Adults (n = 198 410) and Youths (n = 47 625) in the 2011-2012 Data Used to Generate the Percentages and Regression in Table 4 **eTable 8.** Regression Models and Odds Ratios for the Population Divided Into Youth (Age 6-20) and Adult (Age 21 and Older) **eTable 9.** Frequency of All Drugs in 2016 Available to be Assessed in the 2016 Data With the Additional Precision Available Using *ICD-10* and *ICD-10-CM* Codes
