# Supplementary material for: Incidence and Lethality of Suicidal Overdoses by Drug Class
Source: JAMA Netw Open. 2020 Mar 23;3(3):e200607. doi: 10.1001/jamanetworkopen.2020.0607 (PMC7090840; doi:10.1001/jamanetworkopen.2020.0607)
Supplement: Supplement. — eTable 1. Drug Codes for Poisonings of Suicidal and Undetermined Intent, by Coding System eTable 2. Regression Model for 2011-2012 Data Including All Suicide Mechanisms (n = 415 595) eTable 3. Case Fatality Rates (CFR), Case Counts of Suicidal Intent-Only Drug Poisoning Overdoses, and Unweighted Case Counts for 2012 and 2016 NIS/NEDS Data eTable 4. Percent of Cases in the 2011-2012 Overdoses Where a Given Drug Class Was the Only Drug Class Identified eTable 5. Complete Regression Output for Model 1 and Sensitivity Analyses for Models Excluding Unknown Drugs and Excluding Cases of Undetermined Intent eTable 6. Regression Models and Coefficients for Drug Interactions Used to Generate Combined Odds Ratios eTable 7. Drug Frequencies by Setting for Adults (n = 198 410) and Youths (n = 47 625) in the 2011-2012 Data Used to Generate the Percentages and Regression in Table 4 eTable 8. Regression Models and Odds Ratios for the Population Divided Into Youth (Age 6-20) and Adult (Age 21 and Older) eTable 9. Frequency of All Drugs in 2016 Available to be Assessed in the 2016 Data With the Additional Precision Available Using ICD-10 and ICD-10-CM Codes [file jamanetwopen-3-e200607-s001.pdf]

Supplementary Online Content

Miller TR, Swedler DI, Lawrence BA, et al. Incidence and lethality of suicidal overdoses by drug class. *JAMA Netw Open*. 2020;3(3):e200607. doi:10.1001/jamanetworkopen.2020.0607

eTable 1. Drug Codes for Poisonings of Suicidal and Undetermined Intent, by Coding System
eTable 2. Regression Model for 2011-2012 Data Including All Suicide Mechanisms (n = 415 595)
eTable 3. Case Fatality Rates (CFR), Case Counts of Suicidal Intent-Only Drug Poisoning Overdoses, and Unweighted Case Counts for 2012 and 2016 NIS/NEDS Data
eTable 4. Percent of Cases in the 2011-2012 Overdoses Where a Given Drug Class Was the Only Drug Class Identified
eTable 5. Complete Regression Output for Model 1 and Sensitivity Analyses for Models Excluding Unknown Drugs and Excluding Cases of Undetermined Intent
eTable 6. Regression Models and Coefficients for Drug Interactions Used to Generate Combined Odds Ratios
eTable 7. Drug Frequencies by Setting for Adults (n = 198 410) and Youths (n = 47 625) in the 2011-2012 Data Used to Generate the Percentages and Regression in Table 4
eTable 8. Regression Models and Odds Ratios for the Population Divided Into Youth (Age 6-20) and Adult (Age 21 and Older)
eTable 9. Frequency of All Drugs in 2016 Available to be Assessed in the 2016 Data With the Additional Precision Available Using *ICD-10* and *ICD-10-CM* Codes

This supplementary material has been provided by the authors to give readers additional information about their work.

©2020 Miller TR et al. *JAMA Network Open*.

eTable 1. Drug Codes for Poisonings of Suicidal and Undetermined Intent, by Coding System

	2011-16 MCOD: ICD10		2016 HCUP: ICD10-CM		
Drug	Code	Label	Self-inflicted	Undetermined	Label
Anti-diabetic	T38.3	insulin and oral hypoglycemic [antidiabetic] drugs	T38.3X2A	T38.3X4A	insulin and oral hypoglycemic [antidiabetic] drugs
Aspirin	T39.0	salicylates	T39.012A	T39.014A	aspirin
			T39.092A	T39.094A	other salicylates
Acetaminophen	T39.1	4-Aminophenol derivatives	T39.1X2A	T39.1X4A	4-Aminophenol derivatives
Ibuprofen	T39.3	other NSAID	T39.312A	T39.314A	propionic acid derivatives
			T39.392A	T39.394A	other NSAID
Other analgesics*	T39*	nonopioid analgesics, antipyretics and antirheumatics	T39*	T39*	nonopioid analgesics, antipyretics and antirheumatics
Cocaine	T40.5	cocaine	T40.5X2A	T40.5X4A	cocaine
Opioids	T40.0	opium	T40.0X2A	T40.0X4A	opium
	T40.1	heroin	T40.1X2A	T40.1X4A	heroin
	T40.2	other opioids	T40.2X2A	T40.2X4A	other opioids
	T40.3	methadone	T40.3X2A	T40.3X4A	methadone
	T40.4	other synthetic narcotics	T40.4X2A	T40.4X4A	other synthetic narcotics
	T40.6	other and unspecified narcotics	T40.602A	T40.604A	unspecified narcotics
			T40.692A	T40.694A	other narcotics
Hallucinogen	T40.7	cannabis	T40.7X2A	T40.7X4A	Cannabis
	T40.8	LSD	T40.8X2A	T40.8X4A	LSD
	T40.9	other and unspecified hallucinogens	T40.902A	T40.904A	unspecified hallucinogens
			T40.992A	T40.994A	other hallucinogens
Barbiturate	T42.3	barbiturates	T42.3X2A	T42.3X4A	Barbiturates
Benzodiazepine	T42.4	benzodiazepines	T42.4X2A	T42.4X4A	Benzodiazepines
Anti-epileptics	T42.6	Other antiepileptic and sedative-hypnotic drugs	T42.6X2A	T42.6X4A	Other antiepileptic and sedative-hypnotic drugs
Anti-Parkinson’s	T42.8	Antiparkinsonism drugs and other central muscle-tone depressants	T42.8X2A	T42.8X4A	Antiparkinsonism drugs and other central muscle-tone depressants
Antidepressant	T43.0	Tricyclic & tetracyclic antidepressants	T43.012A	T43.014A	tricyclic antidepressants
	T43.1	monoamine-oxidase-inhibitor antidepressants	T43.022A	T43.024A	tetracyclic antidepressants
	T43.2	other and unspecified antidepressants	T43.1X2A	T43.1X4A	monoamine-oxidase-inhibitor antidepressants
			T43.202A	T43.204A	unspecified antidepressants
			T43.212A	T43.214A	selective serotonin & norepinephrine reuptake inhibitors
			T43.222A	T43.224A	selective serotonin reuptake inhibitors
			T43.292A	T43.294A	other antidepressants

©2020 Miller TR et al. *JAMA Network Open*.

Tranquilizer	T43.3	phenothiazine antipsychotics and neuroleptics	T43.3X2A	T43.3X4A	phenothiazine antipsychotics & neuroleptics
	T43.4	butyrophenone and thioxanthene neuroleptics	T43.4X2A	T43.4X4A	butyrophenone and thiothixene neuroleptics
	T43.5	other and unspecified antipsychotics and neuroleptics	T43.502A	T43.504A	unspecified antipsychotics and neuroleptics
			T43.592A	T43.594A	other antipsychotics and neuroleptics
Psychostimulant	T43.6	psychostimulants with abuse potential	T43.602A	T43.604A	unspecified psychostimulants
			T43.622A	T43.624A	Amphetamines
			T43.632A	T43.634A	Methylphenidate
			T43.692A	T43.694A	other psychostimulants
Beta blockers	T44.7	beta-adrenoreceptor antagonists	T44.7X2A	T44.7X4A	beta-adrenoreceptor antagonists
Anti-allergic/ emetic	T45.0	antiallergic and antiemetic drugs	T45.0X2A	T45.0X4A	antiallergic and antiemetic drugs
Calcium channel blockers	T46.1	calcium-channel blockers	T46.1X2A	T46.1X4A	calcium-channel blockers
Muscle relaxants	T48.1	Skeletal muscle relaxants [neuromuscular blocking agents]	T48.1X2A	T48.1X4A	Skeletal muscle relaxants [neuromuscular blocking agents]
Unknown	T50.9	other and unspecified drugs, medicaments and biological substances	T50.902A	T50.904A	unspecified drugs, medicaments and biological substances
			T50.992A	T50.994A	other drugs, medicaments and biological substances
Other specified	All other in T36.0 to T50.9	poisoning by drugs, medicaments and biological substances	All other T36.0 to T50.9 with 6th and 7th digits 2A	All other T36.0 to T50.9 with 6th and 7th digits 4A	poisoning by drugs, medicaments and biological substances
Alcohol	T51.0	ethanol	T51.0X2A	T51.0X4A	Ethanol
	T51.9	alcohol, unspecified	T51.92XA	T51.94XA	unspecified alcohol

In ICD10, intent is coded in underlying cause code. A case must have an underlying cause code between X60-X64 for suicide and Y10-Y14 for undetermined-intent drug poisoning.
In ICD10-CM, intent is coded in the 6th digit of the diagnosis code. Self-inflicted is identified with 6th digit=2, and undetermined is identified with 6th digit=4. All cases are restricted to initial encounter (7th digit=A).
*For Other Analgesics, we selected the remaining codes under the broad T39 category after acetaminophen, aspirin, and ibuprofen were identified. The intentional/unintentional codes under ICD-10-CM would reflect a similar pattern for all the above pain relievers

©2020 Miller TR et al. *JAMA Network Open*.

eTable 1. Drug codes for poisonings of suicidal and undetermined intent, by coding system (continued)

			2011-2012 HCUP: ICD9-CM	
			Codes	Label
Anti-diabetic			962.3	insulins and antidiabetic agents
Anti-allergic/ emetic			963.0	antiallergic and antiemetic drugs
			965.1	Salicylates
			965.4	Aromatic analgesics, not elsewhere classified
			965.61*	Propionic acid derivatives
Other analgesics			965.9	unspecified analgesic and antipyretic
			968.5	Surface [topical] and infiltration anesthetics
			970.81*	Cocaine
			965.00	Opium
			965.01	Heroin
			965.02	Methadone
			965.09	Other opiates and related narcotics
Anti-epileptics			967.8	Other sedatives and hypnotics
Anti-Parkinson’s			968.0	Central nervous system muscle-tone depressants
			969.6	Hallucinogens
			967.0	Barbiturates
			969.4	Benzodiazepine-based tranquilizers
			969.00*	Antidepressant, unspecified
			969.01*	Monoamine oxidase inhibitors
			969.02*	Selective serotonin and norepinephrine reuptake inhibitors
			969.03*	Selective serotonin reuptake inhibitors
			969.04*	Tetracyclic antidepressants
			969.05*	Tricyclic antidepressants
			969.09*	Other antidepressants
			969.1	Phenothiazine-based tranquilizers
			969.2	Butyrophenone-based tranquilizers
			969.3	Other antipsychotics, neuroleptics, and major tranquilizers
			969.4	Benzodiazepine-based tranquilizers
			969.5	Other tranquilizers
			969.70*	Psychostimulant, unspecified
			969.72*	Amphetamines
			969.73*	Methylphenidate
			969.79*	Other psychostimulants
			963.0	Antiallergic and antiemetic drugs
Muscle relaxants			975.2	skeletal muscle relaxants

			977.8	Other specified drugs and medicinal substances
			977.9	Unspecified drug or medicinal substance
			979.9	Other and unspecified vaccines and biological substances
			Any unlisted code 960.0-979.9	poisoning by drugs, medicinal and biological substances
			980.0	ethyl alcohol
			980.9	unspecified alcohol

In ICD9 and ICD9-CM, intent is coded in external cause-of-injury codes (E codes). E codes between E950.0 and E950.5 identify suicide/self-inflicted; E codes between E980.0 and E980.5 identify undetermined intent.

©2020 Miller TR et al. *JAMA Network Open*.

eTable 2. Regression Model for 2011-2012 Data Including All Suicide Mechanisms (n = 415 595)

	ORs	95% LB	95% UB
**Opioid**	7.24	6.67	7.79
**4-Aminophenol derivative**	1.02	0.90	1.19
**Anti-allergy/anti-emetic**	4.91	4.35	5.70
**Anti-depressant**	3.89	3.53	4.27
**Anti-diabetic**	3.06	2.26	4.07
**Anti-epileptic**	1.38	1.18	1.59
**Anti-Parkinson**	0.75	0.59	0.98
**Salicylates**	1.81	1.33	2.38
**Barbiturate**	4.83	3.72	6.31
**Benzodiazepine**	0.82	0.75	0.89
**Cocaine**	1.59	1.31	1.93
**Hallucinogen**	0.22	0.13	0.38
**Ibuprofen**	0.25	0.17	0.38
**Muscle relaxants**	0.05	0.02	0.15
**Other analgesics**	0.56	0.23	1.47
**Stimulant**	1.69	1.36	2.09
**Other Tranquilizers**	1.71	1.51	2.01
**Alcohol**	2.47	2.22	2.73
**Other named drug**	1.37	0.95	1.19
**Unknown drug**	4.40	3.92	4.85
**AZ**	1.00		
**CA**	1.37	1.24	1.54
**FL**	1.29	1.17	1.42
**IA**	0.59	0.45	0.74
**KY**	1.46	1.30	1.65
**NC**	1.34	1.20	1.49
**NE**	1.74	1.38	2.17
**NJ**	0.90	0.80	1.01
**NY**	1.01	0.90	1.09
**RI**	0.79	0.65	0.97
**UT**	2.50	2.17	2.92
**Male gender**	1.72	1.56	2.16
**Age 6 - 14**	0.38	0.27	0.53
**Age 15 - 20**	1.00		
**Age 21 - 25**	2.24	1.85	2.72
**Age 26 - 30**	3.35	2.78	4.05
**Age 31 - 39**	5.31	4.49	6.28
**Age 40 - 49**	11.81	10.10	13.81
**Age 50 - 59**	19.90	16.99	23.29
**Age 60+**	30.37	25.85	35.68

©2020 Miller TR et al. *JAMA Network Open*.

**Male*Age 6 - 14**	0.88	0.59	1.33
**Male*Age 15 - 20**	1.00		
**Male*Age 21 - 25**	0.83	0.66	1.04
**Male*Age 26 - 30**	0.76	0.61	0.95
**Male*Age 31 - 39**	0.78	0.64	0.95
**Male*Age 40 - 49**	0.70	0.58	0.85
**Male*Age 50 - 59**	0.84	0.70	1.01
**Male*Age 60+**	0.94	0.77	1.13
**2012 case**	1.07	1.02	1.12
**Comorbidity**	0.03	0.03	0.03
**Large urban**	1.00		
**Small urban**	1.14	1.09	1.20
**Rural**	0.90	0.84	0.97
**Mechanism- Cutting**	1.00		
**Mechanism- Drowning**	176.53	140.43	221.91
**Mechanism- Fall**	19.57	17.10	22.40
**Mechanism- Fire**	3.42	2.78	4.22
**Mechanism- Firearm**	164.64	147.63	183.61
**Mechanism- Hot object/vapors***	1.00		
**Mechanism- Motor vehicle***	1.00		
**Mechanism- Nature***	1.00		
**Mechanism- Other**	1.23	1.08	1.40
**Mechanism- Poisoning**	1.04	0.92	1.17
**Mechanism- Poisoning (non-drugs)**	3.42	3.02	3.87
**Mechanism- Suffocation**	179.01	160.04	200.23
**Mechanism- Transportation**	925.79	448.23	1912.16
**Mechanism- Unspecified**	0.56	0.49	0.65

*Three mechanisms (Hot objects/vapors; motor vehicle; nature) perfectly predicted the outcome in regression modeling. These variables were excluded, and their cases were pooled into the base with Mechanism- Cutting.

©2020 Miller TR et al. *JAMA Network Open*.

eTable 3. Case Fatality Rates (CFR), Case Counts of Suicidal Intent-Only Drug Poisoning Overdoses, and Unweighted Case Counts for 2012 and 2016 NIS/NEDS Data

	CFR, 2011-12	CFR 2012	CFR 2016	Fatal, No Undetermined, 2011-12	Admitted, No Undetermined, 2011-12	ED, No Undetermined, 2011-12	Admitted Unweighted 2012	ED Unweighted 2012	Admitted Unweighted 2016	ED Unweighted 2016
**Cases**	2.1%	2.2%	3.4%	3,870	92,669	70,424	51,780	41,473	35,012	27,832
**Male Cases**	2.6%	2.7%	4.8%	2,026	35,666	25,752	22,789	17,019	13,312	10,594
**Female Cases**	1.7%	1.8%	2.6%	1,844	57,003	44,672	28,991	24,454	21,700	17,238
**Age 6-14**	0.1%	0.1%	0.2%	2	2,236	3,952	2,405	953	2,556	848
**Age 15-20**	0.3%	0.3%	0.5%	86	12,143	16,972	10,377	4,865	8,910	4,032
**Age 21-25**	0.9%	0.9%	1.6%	160	9,852	9,417	7,165	4,094	4,666	2,977
**Age 26-30**	1.2%	1.5%	2.5%	210	9,018	7,594	5,993	3,895	3,817	2,618
**Age 31-39**	1.7%	1.9%	3.4%	461	15,554	11,262	8,373	7,699	5,351	4,645
**Age 40-49**	2.7%	3.0%	4.6%	941	20,189	11,457	8,323	8,454	4,589	4,991
**Age 50-59**	3.9%	4.4%	6.6%	1,146	15,105	7,106	5,571	7,288	3,491	4,609
**Age 60+**	4.8%	4.8%	9.0%	864	8,513	2,623	2,767	4,146	1,632	3,112
			0.2%							
**Undetermined intent**	1.5%	2.1%		0	0	0	21,560	8,811	7,049	3,146
**Alcohol-involved**	3.3%	4.2%	7.3%	399	6,732	3,027	2,509	2,907	1,579	2,216
**Drug classes per case[b]**				1.34 (0.02)	1.43 (0.003)	1.19 (0.002)	1.06 (0.002)	1.41 (0.004)	1.21 (0.003)	1.45 (0.005)
**4-Aminophenol derivative**	1.0%	0.9%	1.1%	241	14,252	7,793	3,711	5,390	3,201	3,871
**Anti-allergy/anti-emetic**	3.8%	3.1%	4.4%	317	4,843	3,460	1,900	2,116	1,925	1,845
**Anti-depressant**	3.1%	2.9%	3.6%	674	15,371	8,794	4,655	6,489	4,767	4,848
**Anti-diabetic**	1.7%	1.4%	2.3%	51	2,012	543	378	880	279	734
**Anti-epileptic**	2.4%	2.2%	2.8%	238	6,034	3,558	2,040	2,429	2,150	2,792
**Anti-Parkinson's**	1.5%	1.6%	2.1%	67	2,327	1,567	938	955	478	727
**Salicylates**	1.1%	1.0%	1.0%	56	2,961	1,665	782	1,217	623	766
**Barbiturate**	6.1%	5.8%	12.2%	80	834	184	133	373	60	173
**Benzodiazepine**	1.5%	1.3%	2.6%	586	27,375	14,700	8,875	11,740	5,660	6,604
**Cocaine**	1.3%	1.8%	7.7%	69	2,331	789	1,059	1,640	455	717
**Hallucinogen**	0.3%	0.4%	0.6%	4	882	485	1,060	615	557	398
**Ibuprofen**	0.2%	0.2%	0.2%	24	4,574	6,370	3,091	1,736	3,359	1,703
**Muscle relaxants**	0.1%	0.1%	4.0%	3	2,375	989	613	1,101	454	623

©2020 Miller TR et al. *JAMA Network Open*.

**Opioid**	5.2%	4.5%	9.5%	1,250	12,388	6,912	6,650	8,068	5,875	4,327
**Other analgesics**	0.6%	0.6%	0.4%	5	288	317	170	102	117	76
**Other tranquilizer**	1.6%	1.6%	2.2%	220	9,934	4,970	2,563	3,850	2,670	3,394
**Psychostimulant**	1.8%	1.6%	3.5%	60	2,072	1,287	1,410	1,408	1,283	1,127
**Other named drug**	1.4%	1.2%	1.8%	490	18,500	10,778	7,131	8,181	4,333	4,085
**Unknown drug only**	3.6%	3.7%	5.5%	737	3,506	8,463	6,053	1,304	3,814	1,143

[a] Case fatality rate for a given drug is calculated as percent of cases that were fatalities within total cases determined to have that drug. CFR_i = ($Fatalities_i$/Total $Cases_i$)

[b] Drug classes per case presented as mean and standard error in parentheses.

©2020 Miller TR et al. *JAMA Network Open*.

eTable 4. Percent of Cases in the 2011-2012 Overdoses Where a Given Drug Class Was the Only Drug Class Identified

Drug Class	**Percent for fatal cases**	**Percent for non-fatal cases**
4-Aminophenol derivative	32.7%	58.1%
Anti-allergy/Anti-emetic	34.0%	51.2%
Anti-depressant	31.7%	46.1%
Anti-diabetic	81.8%	66.9%
Anti-epileptic	14.9%	46.2%
Anti-Parkinson’s	13.6%	43.2%
Salicylates	60.3%	57.9%
Barbiturate	37.9%	33.7%
Benzodiazepine	11.8%	48.6%
Cocaine	16.2%	63.9%
Hallucinogens	15.4%	49.9%
Ibuprofen	7.7%	55.5%
Muscle Relaxants	33.3%	38.3%
Opioids	37.0%	53.5%
Other Analgesics	40.0%	62.4%
Other Tranquilizer	23.0%	47.0%
Psychostimulants	31.9%	53.0%
Other Named Drug	62.5%	55.8%
Unknown Drug Only	N/A	N/A

©2020 Miller TR et al. *JAMA Network Open*.

eTable 5. Complete Regression Output for Model 1 and Sensitivity Analyses for Models Excluding Unknown Drugs and Excluding Cases of Undetermined Intent

Model	Model 1			Excluding Unknown Drugs			Excluding Undetermined Intent		
	OR	95% LB	95% UB	OR	95% LB	95% UB	OR	95% LB	95% UB
**Opioid**	5.55	5.15	5.97	4.70	4.35	5.08	4.22	3.84	4.63
**4-Aminophenol derivative**	0.94	0.82	1.09	1.01	0.88	1.17	0.63	0.54	0.73
**Anti-allergy/anti-emetic**	4.19	3.66	4.79	4.32	3.77	4.94	2.80	2.41	3.24
**Anti-depressant**	3.37	3.07	3.70	3.54	3.22	3.89	1.99	1.79	2.21
**Anti-diabetic**	2.66	1.98	3.59	2.89	2.14	3.90	2.19	1.58	3.04
**Anti-epileptic**	1.21	1.05	1.40	1.29	1.11	1.49	0.83	0.71	0.98
**Anti-Parkinson's**	0.67	0.52	0.87	0.76	0.59	0.97	0.64	0.48	0.85
**Salicylates**	1.61	1.08	1.60	1.65	1.23	2.21	1.16	0.85	1.57
**Benzodiazepine**	0.71	0.65	0.78	5.08	3.87	6.66	0.43	0.39	0.48
**Barbiturate**	4.60	3.52	6.01	0.82	0.75	0.90	4.26	3.20	5.61
**Cocaine**	1.31	1.08	1.60	1.40	1.14	1.70	1.09	0.82	1.45
**Hallucinogen**	0.20	0.11	0.35	0.20	0.12	0.36	0.18	0.06	0.49
**Ibuprofen**	0.24	0.16	0.35	0.24	0.16	0.36	0.16	0.11	0.25
**Muscle relaxant**	0.04	0.01	0.14	0.05	0.01	0.14	0.05	0.01	0.14
**Other analgesic**	0.46	0.18	1.16	0.47	0.18	1.18	0.42	0.16	1.06
**Other tranquilizer**	1.52	1.32	1.76	1.56	1.35	1.80	0.99	0.85	1.17
**Stimulant**	1.45	1.17	1.80	1.52	1.23	1.89	1.23	0.90	1.67
**Alcohol**	2.08	1.67	2.32	1.95	1.73	2.18	2.05	1.71	2.35
**Other named drug**	1.18	1.07	1.31	0.47	1.11	1.36	1.05	0.94	1.18
**Unknown drug only**	3.03	2.73	3.35				1.94	1.71	2.21
**AZ**	1.00			1.00			1.00		
**CA**	1.31	1.12	1.53	1.35	1.12	1.63	1.79	1.46	2.19
**FL**	1.54	1.34	1.78	1.80	1.53	2.12	2.25	1.88	2.67
**IA**	0.38	0.24	0.61	0.50	0.31	0.80	0.34	0.18	0.65
**KY**	1.16	0.96	1.39	1.21	0.98	1.50	1.28	1.00	1.64
**NE**	1.85	1.32	2.60	1.86	1.55	2.24	2.33	1.90	2.86
**NC**	1.51	1.29	1.78	1.96	1.35	2.86	2.81	1.91	4.14
**NJ**	0.83	0.70	0.99	0.86	0.70	1.05	1.77	1.42	2.21
**NY**	0.98	0.85	1.13	1.31	1.11	1.54	1.44	1.19	1.74
**RI**	0.62	0.44	0.86	0.82	0.58	1.16	0.76	0.52	1.13
**UT**	3.15	2.59	3.82	3.80	3.08	4.69	2.62	2.01	3.43
**Male**	4.06	2.70	6.12	4.38	2.85	6.74	4.92	3.08	7.84
**Age 6 - 14**	0.51	0.20	1.30	0.57	0.22	1.47	0.25	0.06	1.05
**Age 15 - 20**	1.00			1.00			1.00		
**Age 21 - 25**	4.86	3.24	7.28	4.41	2.86	6.78	5.61	3.55	8.87
**Age 26 - 30**	8.08	5.46	11.95	7.17	4.73	10.88	9.22	5.90	14.39
**Age 31 - 39**	13.98	9.70	20.15	11.74	7.96	17.31	16.64	11.00	25.18
**Age 40 - 49**	33.13	23.23	48.46	27.13	18.64	39.49	40.10	26.85	59.90
**Age 50 - 59**	56.44	39.56	80.52	46.59	32.00	67.82	83.14	55.66	124.18
**Age 60+**	92.61	64.67	132.64	77.08	52.69	112.75	184.98	123.11	277.93
**Male*Age 6 - 14**	0.50	0.12	1.97	0.51	0.13	2.04	--		
**Male*Age 15 - 20**	1.00			1.00			1.00		
**Male*Age 21 - 25**	0.47	0.29	0.76	0.51	0.30	0.85	0.40	0.23	0.71
**Male*Age 26 - 30**	0.40	0.25	0.64	0.40	0.24	0.66	0.43	0.25	0.74
**Male*Age 31 - 39**	0.41	0.26	0.64	0.44	0.27	0.70	0.40	0.24	0.67
**Male*Age 40 - 49**	0.33	0.21	0.50	0.35	0.22	0.55	0.35	0.21	0.57
**Male*Age 50 - 59**	0.34	0.22	0.52	0.35	0.22	0.56	0.35	0.22	0.57
**Male*Age 60+**	0.28	0.18	0.44	0.30	0.19	0.47	0.29	0.17	0.47
**2012 case**	1.05	0.97	1.13	1.05	0.97	1.15	1.06	0.97	1.16

©2020 Miller TR et al. *JAMA Network Open*.

**Comorbidity**	0.03	0.02	0.03	0.02	0.02	0.03	0.02	0.01	0.02
**Large urban**	1.00			1.00			1.00		
**Small urban**	1.16	1.08	1.25	1.20	1.11	1.31	0.98	0.90	1.07
**Rural**	0.89	0.79	0.99	0.92	0.81	1.04	0.75	0.65	0.86

©2020 Miller TR et al. *JAMA Network Open*.

eTable 6. Regression Models and Coefficients for Drug Interactions Used to Generate Combined Odds Ratios

Model	Opioid Interaction Model			Alcohol Interaction Model		
	Coeff.	95% LB	95% UB	Coeff.	95% LB	95% UB
**Opioid**	1.42	1.32	1.52	1.62	1.54	1.70
**4-Aminophenol derivative**	-0.23	-0.42	-0.05	-0.07	-0.22	0.08
**Anti-allergy/anti-emetic**	1.31	1.16	1.46	1.32	1.17	1.46
**Anti-depressant**	1.12	1.01	1.23	1.14	1.04	1.24
**Anti-diabetic**	0.89	0.59	1.19	0.94	0.64	1.24
**Anti-epileptic**	0.04	-0.14	0.23	0.11	-0.05	0.26
**Anti-Parkinson**	-0.46	-0.71	-0.20	-0.41	-0.66	-0.16
**Salicylates**	0.42	0.12	0.71	0.44	0.15	0.73
**Benzodiazepine**	-0.82	-0.95	-0.69	-0.43	-0.53	-0.34
**Barbiturate**	1.47	1.20	1.74	1.53	1.27	1.80
**Cocaine**	0.06	-.021	0.34	0.28	0.07	0.49
**Hallucinogen**	-1.68	-2.25	-1.11	-1.61	-2.18	-1.04
**Ibuprofen**	-1.48	-1.88	-1.08	-1.45	-1.85	-1.05
**Muscle relaxants**	-3.16	-4.30	-2.01	-3.11	-4.25	-1.97
**Other analgesics**	-0.91	-1.86	0.04	-0.85	-1.81	0.11
**Psychostimulants**	0.42	0.16	0.68	0.37	0.12	0.57
**Other tranquilizer**	0.31	0.15	0.48	0.37	0.22	0.52
**Alcohol**	0.55	0.41	0.68	0.04	-0.15	0.22
**Other named drug**	0.21	0.10	0.33	0.14	0.04	0.25
**Unknown drug**	0.96	0.85	1.07	0.97	0.87	1.08
**AZ**	0.00			0.00		
**CA**	0.27	0.11	0.43	0.26	0.10	0.41
**FL**	0.41	0.27	0.55	0.43	0.29	0.57
**IA**	-0.97	-1.44	-0.51	-0.97	-1.43	-0.50
**KY**	0.13	-0.06	0.31	0.14	-0.04	0.32
**NE**	0.61	0.24	0.57	0.59	0.24	0.93
**NC**	0.40	0.27	0.95	0.40	0.24	0.57
**NJ**	-0.18	-0.35	-0.00	-0.15	-0.32	0.02
**NY**	-0.05	-0.20	0.09	-0.03	-0.17	0.12
**RI**	-0.47	-0.80	-0.14	-0.47	-0.80	-0.14
**UT**	1.15	0.96	1.35	1.15	0.96	1.34
**Male**	1.39	0.98	1.80	1.40	1.00	1.81
**Age Group†**	--					
**Age Group*Male†**	--					
**2012 case**	0.05	-0.02	0.13	0.03	-0.04	0.11
**Comorbidity**	-3.64	-3.73	-3.56	-3.65	-3.73	-3.56
**Large urban**	0.00			0.00		
**Small urban**	0.15	0.08	0.23	0.15	0.08	0.23
**Rural**	-0.13	-0.24	-0.02	-0.11	-0.22	0.01
Drug of interest interacted with each of the below substances						
**Opioid**	--			0.75	0.55	1.00
**4-Aminophenol derivative**	0.31	0.01	0.60	-0.08	-0.54	0.39
**Anti-allergy/anti-emetic**	0.43	0.07	0.79	0.85	0.44	1.26
**Anti-depressant**	0.26	0.04	0.48	0.60	0.30	0.90
**Anti-epileptic**	0.33	0.01	0.65	0.60	0.16	1.05
**Benzodiazepine**	0.96	0.77	1.14	0.64	0.37	0.90
**Cocaine**	0.35	-0.06	0.74	-0.23	-0.82	0.36
**Stimulant**	-0.35	-0.80	0.11	0.20	-0.50	0.99
**Other tranquilizer**	0.40	0.06	0.75	0.50	0.03	0.97
**Other named drug**	-0.84	-1.14	-0.59	-0.22	-0.63	0.19
**Alcohol**	0.52	0.28	0.76	--		
**Unknown drug**	--			2.05	1.55	2.55

†Age group and age group by male gender results excluded for readability

©2020 Miller TR et al. *JAMA Network Open*.

eTable 6. Regression models and coefficients for drug interactions used to generate combined odds ratios. (continued)

Model	4-Aminophenol derivative Interaction Model			Anti-depressant Interaction Model		
	Coeff.	95% LB	95% UB	Coeff.	95% LB	95% UB
**Opioid**	1.70	1.62	1.78	1.68	1.60	1.76
**4-Aminophenol derivative**	-0.13	-0.34	0.09	0.00	-0.15	0.15
**Anti-allergy/anti-emetic**	1.44	1.30	1.58	1.38	1.23	1.53
**Anti-depressant**	1.24	1.14	1.33	1.08	0.94	1.22
**Anti-diabetic**	0.98	0.68	1.27	1.00	0.70	1.30
**Anti-epileptic**	0.18	0.03	0.33	0.03	-0.15	0.21
**Anti-Parkinson**	-0.39	-0.64	-0.14	-0.41	-0.66	-0.16
**Salicylates**	0.48	0.19	0.77	0.46	0.17	0.75
**Benzodiazepine**	-0.36	-0.45	-0.27	-0.40	-0.50	-0.29
**Barbiturate**	1.53	1.26	1.80	1.53	1.26	1.80
**Cocaine**	0.28	0.08	0.48	0.21	-0.01	0.42
**Hallucinogen**	-1.62	-2.19	-1.05	-1.62	-2.19	-1.05
**Ibuprofen**	-1.44	-1.84	-1.04	-1.45	-1.85	-1.05
**Muscle relaxant**	-3.14	-4.29	-2.00	-3.15	-4.29	-2.01
**Other analgesics**	-0.77	-1.70	0.16	-0.81	-1.85	0.14
**Stimulant**	0.39	0.17	0.61	0.27	0.03	0.51
**Other tranquilizer**	0.40	0.26	0.55	0.47	0.29	0.64
**Alcohol**	0.73	0.62	0.84	0.64	0.52	0.77
**Other named drug**	0.19	0.09	0.30	0.29	0.18	0.40
**Unknown drug**	1.11	1.00	1.21	1.08	0.98	1.19
**AZ**	0.00			0.00		
**CA**	0.26	0.11	0.42	0.26	0.10	0.42
**FL**	0.43	0.29	0.57	0.43	0.29	0.57
**IA**	-0.97	-1.44	-0.51	-0.98	-1.44	-0.52
**KY**	0.15	-0.04	0.33	0.14	-0.04	0.33
**NE**	0.62	0.27	0.96	0.61	0.27	0.95
**NC**	0.41	0.25	0.57	0.41	0.25	0.57
**NJ**	-0.19	-0.36	-0.01	-0.18	-0.36	-0.01
**NY**	-0.03	-0.17	0.12	-0.04	-0.18	0.11
**RI**	-0.48	-0.81	-0.15	-0.49	-0.82	-0.16
**UT**	1.14	0.95	1.34	1.14	0.95	1.34
**Male**	1.40	0.99	1.81	1.40	0.99	1.80
**AgeGroup†**						
**Age*Male†**						
**2012 case**	0.04	-0.04	0.12	0.05	-0.03	0.12
**Comorbidity**	-3.64	-3.72	-3.55	-3.64	-3.73	-3.56
**Large urban**	0.00			0.00		
**Small urban**	0.15	0.08	0.22	0.15	0.08	0.23
**Rural**	-0.12	-0.23	-0.01	-0.12	-0.23	-0.01
Drug of interest interacted with each of the below substances						
**Opioid**	0.32	0.01	0.62	0.32	0.10	0.54
**4-Aminophenol derivative**	--			-0.62	-1.08	-0.15
**Anti-allergy/anti-emetic**	0.03	-0.9	0.44	0.28	-0.07	0.63
**Anti-depressant**	-0.54	-1.02	-0.07	--		
**Anti-epileptic**	0.19	-0.39	0.72	0.57	0.24	0.89
**Benzodiazepine**	0.26	-0.12	0.63	0.21	-0.01	0.44
**Cocaine**	-0.41	-1.74	0.92	0.62	0.01	1.22
**Stimulant**	-0.34	-1.46	0.78	0.58	0.01	1.16
**Other tranquilizer**	0.46	-0.27	1.28	-0.08	-0.38	0.23
**Other named drug**	-0.64	-1.16	-0.12	-0.88	-1.17	-0.58
**Alcohol**	0.07	-0.38	0.50	0.52	0.24	0.80

†Age group and age group by male gender results excluded for readability

©2020 Miller TR et al. *JAMA Network Open*.

eTable 6. Regression models and coefficients for drug interactions used to generate combined odds ratios. (continued)

Model	Anti-epileptics Interaction Model			Benzodiazepine Interaction Model		
	**Coeff.**	**95% LB**	**95% UB**	**Coeff.**	**95% LB**	**95% UB**
**Opioid**	1.67	1.60	1.75	1.45	1.37	1.54
**4-Aminophenol derivative**	-0.07	-0.22	0.07	-0.12	-0.28	0.03
**Anti-allergy/anti-emetic**	1.38	1.24	1.52	1.20	1.05	1.35
**Anti-depressant**	1.17	1.07	1.27	1.09	0.98	1.19
**Anti-diabetic**	0.96	0.66	1.26	0.88	0.58	1.18
**Anti-epileptic**	-0.39	-0.64	-0.14	-0.01	-0.17	0.18
**Anti-Parkinson**	-0.40	-0.65	-0.15	-0.45	-0.70	-0.17
**Salicylates**	0.46	0.17	0.75	0.39	0.10	0.69
**Benzodiazepine**	-0.39	-0.48	-0.29	-1.21	-1.38	-1.05
**Barbiturate**	1.53	1.26	1.80	1.49	1.22	1.76
**Cocaine**	0.27	0.07	0.47	0.08	-0.15	0.31
**Hallucinogen**	-1.62	-2.19	-1.06	-1.72	-2.28	-1.14
**Ibuprofen**	-1.46	-1.86	-1.06	-1.48	-1.88	-1.08
**Muscle relaxant**	-3.18	-4.32	-2.03	-3.20	-4.35	-2.05
**Other analgesic**	-0.80	-1.73	0.13	-0.93	-1.88	0.02
**Stimulant**	0.34	0.12	0.56	0.41	0.18	0.65
**Other tranquilizer**	0.32	0.17	0.47	0.26	0.09	0.42
**Alcohol**	0.69	0.57	0.80	0.54	0.42	0.67
**Other named drug**	0.17	0.06	0.27	0.11	-0.00	0.22
**Unknown drug**	1.06	0.96	1.16	0.92	0.82	1.03
**AZ**	0.00			0.00		
**CA**	0.27	0.11	0.43	0.26	0.10	0.42
**FL**	0.43	0.29	0.57	0.42	0.28	0.56
**IA**	-0.96	-1.42	-0.50	-0.97	-1.43	-0.51
**KY**	0.15	-0.04	0.33	0.13	-0.06	0.31
**NE**	0.63	0.29	0.97	0.62	0.28	0.97
**NC**	0.41	0.25	0.57	0.40	0.24	0.57
**NJ**	-0.19	-0.36	-0.02	-0.19	.0.36	-0.02
**NY**	-0.03	-0.17	0.11	-0.05	-0.19	0.10
**RI**	-0.48	-0.81	-0.15	-0.49	-0.82	-0.16
**UT**	1.14	0.95	1.33	1.17	0.98	1.36
**Male**	1.40	0.99	1.81	1.39	0.98	1.80
**AgeGroup†**	--			--		
**Age*Male†**	--			--		
**2012 case**	0.04	-0.03	0.12	0.05	-0.03	0.13
**Comorbidity**	-3.64	-3.72	-3.55	-3.63	-3.72	-3.54
**Large urban**	0.00			0.00		
**Small urban**	0.15	0.08	0.23	0.15	0.08	0.23
**Rural**	-0.12	-0.23	-0.01	-0.12	-0.23	-0.01
Drug of interest interacted with each of the below substances						
**Opioid**	0.43	0.10	0.76	1.16	0.96	1.35
**4-Aminophenol derivative**	-0.01	-0.69	0.49	0.01	-0.36	0.40
**Anti-allergy/anti-emetic**	0.65	0.10	1.19	1.08	0.74	1.46
**Anti-depressant**	0.44	0.09	0.78	0.39	0.18	0.62
**Anti-epileptic**	--			0.57	0.25	0.91
**Benzodiazepine**	0.48	0.14	0.82	--		
**Cocaine**	-0.19	-1.71	1.33	0.57	0.09	1.03
**Stimulant**	1.00	-0.30	2.30	-0.77	-1.41	-0.14
**Other tranquilizer**	0.90	0.45	1.36	0.67	0.32	1.01
**Other named drug**	-0.62	-1.12	-0.11	-0.27	-0.61	0.06
**Alcohol**	0.70	0.27	1.13	0.76	0.50	1.02

†Age group and age group by male gender results excluded for readability

©2020 Miller TR et al. *JAMA Network Open*.

eTable 6. Regression models and coefficients for drug interactions used to generate combined odds ratios. (continued)

Model	Anti-allergy/anti-emetic Interaction Model		
	Coeff.	95% LB	95% UB
**Opioid**	1.69	1.62	1.77
**4-Aminophenol derivative**	-0.04	-0.20	0.11
**Anti-allergy/anti-emetic**	1.22	1.03	1.41
**Anti-depressant**	1.20	1.10	1.30
**Anti-diabetic**	0.96	0.67	1.26
**Anti-epileptic**	0.14	-0.02	0.29
**Anti-Parkinson**	-0.39	-0.65	-0.15
**Salicylates**	0.46	0.17	0.76
**Benzodiazepine**	-0.40	-0.49	-0.30
**Barbiturate**	1.53	1.26	1.80
**Cocaine**	0.24	0.03	0.44
**Hallucinogen**	-1.62	-2.19	-1.05
**Ibuprofen**	-1.43	-1.83	-1.03
**Muscle relaxant**	-3.18	-4.33	-2.03
**Other analgesic**	-0.78	-1.71	0.15
**Stimulant**	0.31	0.09	0.54
**Other tranquilizer**	0.42	0.27	0.57
**Alcohol**	0.68	0.57	0.80
**Other named drug**	-0.20	0.09	0.30
**Unknown drug**	1.09	0.98	1.18
**AZ**	0.00		
**CA**	0.26	0.10	0.42
**FL**	0.43	0.29	0.57
**IA**	-0.95	-1.41	-0.49
**KY**	0.15	-0.04	0.33
**NE**	0.62	0.28	0.96
**NC**	0.41	0.25	0.57
**NJ**	-0.19	-0.36	-0.02
**NY**	-0.03	-0.18	0.11
**RI**	-0.49	-0.82	-0.16
**UT**	1.14	0.95	1.33
**Male**	1.40	0.99	1.81
**AgeGroup†**	--		
**Age*Male†**	--		
**2012 case**	0.04	-0.03	0.12
**Comorbidity**	-3.64	-3.72	-3.55
**Large urban**	0.00		
**Small urban**	0.15	0.08	0.23
**Rural**	-0.12	-0.23	-0.01
**Opioid**	0.25	-0.12	0.62
**4-Aminophenol derivative**	-0.15	-0.56	0.27
**Anti-allergy/anti-emetic**	--		
**Anti-depressant**	0.03	-0.34	0.40
**Anti-epileptic**	0.56	0.03	1.10
**Benzodiazepine**	0.83	0.46	1.21
**Cocaine**	1.20	0.05	2.35
**Stimulant**	0.99	0.12	1.85
**Other tranquilizer**	-0.04	-0.61	0.53
**Other named drug**	-0.80	-1.27	-0.33
**Alcohol**	0.54	0.14	0.94

†Age group and age group by male gender results excluded for readability

eTable 7. Drug Frequencies by Setting for Adults (n = 198 410) and Youths (n = 47 625) in the 2011-2012 Data Used to Generate the Percentages and Regression in Table 4

Drug	Youth fatalities	Youth admitted	Youth ED	Adult fatalities	Adult admitted	Adult ED
4-Aminophenol derivative	6	3,812	3,840	272	11,893	5,586
Alcohol	1	576	1,146	549	8,044	6,386
Anti-allergy/anti-emetic	11	1,246	1,649	362	3,968	2,581
Anti-depressant	17	2,670	3,154	834	13,893	7,003
Anti-diabetic	1	218	142	54	2,100	702
Anti-epileptic	2	473	681	280	6,329	3,898
Anti-Parkinson's	1	173	245	80	2,705	2,202
Salicylates	4	1,031	1,005	54	2,204	931
Barbiturate	1	65	35	94	1,081	272
Benzodiazepine	10	1,951	2,604	798	32,336	17,762
Cocaine	0	163	175	130	6,833	2,357
Hallucinogen	1	390	1,098	12	1,525	1,326
Ibuprofen	0	1,791	3,802	26	3,003	3,311
Muscle relaxant	0	227	207	3	2,480	1,072
Opioid	45	1,248	2,025	1,936	18,496	14,116
Other analgesic	0	83	169	5	297	257
Other tranquilizer	6	1,522	1,675	276	9,294	4,387
Stimulant	6	669	1,148	110	2,763	1,839
Other named drug	17	3,471	4,758	529	18,950	11,163
Unknown drug only	11	639	4,193	693	2,883	10,847
Drugs per case*	1.13	1.34	1.04	1.32	1.37	1.03

*Drugs per case calculation excludes alcohol.

eTable 8. Regression Models and Odds Ratios for the Population Divided Into Youth (Age 6-20) and Adult (Age 21 and Older)

Model	Youth, Under 21			Adults, 21 and Older		
	OR	95% LB	95% UB	OR	95% LB	95% UB
**4-Aminophenol derivative**	0.43	0.19	1.01	0.97	0.84	1.12
**Alcohol**	0.25	0.03	1.85	2.12	1.90	2.37
**Anti-allergy/anti-emetic**	2.14	1.07	4.28	4.30	3.74	4.93
**Anti-depressant**	2.44	1.38	4.31	3.39	3.08	3.73
**Anti-diabetic**	2.79	0.37	20.91	2.66	1.97	3.59
**Anti-epileptic**	0.77	0.19	3.16	1.22	1.05	1.41
**Anti-Parkinson's**	0.61	0.08	4.51	0.68	0.53	0.87
**Salicylates**	1.61	0.57	4.51	1.60	1.18	2.16
**Barbiturate**	9.64	1.26	73.91	4.60	3.51	6.02
**Benzodiazepine**	0.72	0.36	1.40	0.71	0.65	0.78
**Cocaine**				1.34	1.10	1.63
**Hallucinogen**	0.18	0.03	1.33	0.20	0.11	0.36
**Ibuprofen**				0.26	0.18	0.39
**Opioids**	8.47	5.39	13.28	5.49	5.09	5.91
**Other analgesic**				0.48	0.19	1.22
**Other tranquilizer**	1.11	0.47	2.62	1.53	1.32	1.77
**Psychostimulants**	1.31	0.55	3.11	1.45	1.16	1.81
**Other named drug class**	1.06	0.62	1.84	1.18	1.06	1.31
**Unknown drug only**	0.98	0.48	1.98	3.13	2.82	3.47
**AZ**	1.00			1.00		
**CA**	1.27	0.48	1.98	1.31	1.11	1.53
**FL**	1.45	0.60	3.54	1.54	1.34	1.78
**IA**				0.40	0.25	0.63
**KY**	0.93	0.30	2.85	1.17	0.97	1.40
**NC**	0.40	0.12	1.34	1.56	1.32	1.84
**NE**	1.73	0.35	8.70	1.87	1.32	2.64
**NJ**	1.69	0.63	4.53	0.82	0.69	0.97
**NY**	0.96	0.38	2.40	0.97	0.84	1.13
**RI**	0.72	0.09	6.06	0.62	0.44	0.86
**UT**	4.62	1.77	12.03	3.07	2.52	3.74
**Male**	3.72	2.44	5.66	1.91	1.46	2.50
**Age 6-14**	0.55	0.21	1.41	--		
**Age 15-20**	1.00			--		
**Age 21-25**	--			1.00		
**Age 26-30**	--			1.66	1.25	2.21
**Age 31-39**	--			2.88	2.24	3.87
**Age 40-49**	--			6.84	5.42	8.62
**Age 50-59**	--			11.67	9.25	14.72
**Age 60+**	--			19.16	15.10	24.32
**Male*Age 6 - 14**	0.51	0.13	2.05	--		
**Male*Age 15 - 20**	1.00			--		
**Male*Age 21 - 25**	--			1.00		
**Male*Age 26 - 30**	--			0.84	0.59	1.22
**Male*Age 31 - 39**	--			0.88	0.64	1.21
**Male*Age 40 - 49**	--			0.70	0.52	0.94
**Male*Age 50 - 59**	--			0.73	0.54	0.97
**Male*Age 60+**	--			0.60	0.44	0.82
**2012 case**	0.74			1.05	0.98	1.14
**Comorbidity**	0.04			0.03	0.02	0.03
**Large urban**	1.00			1.00		
**Small urban**	1.40	0.93	2.10	1.15	1.07	1.24
**Rural**	1.18	0.62	2.26	0.88	0.78	0.98

©2020 Miller TR et al. *JAMA Network Open*.

eTable 9. Frequency of All Drugs in 2016 Available to be Assessed in the 2016 Data With the Additional Precision Available Using *ICD-10* and *ICD-10-CM* Codes

	**Fatal 2016**	**Admitted 2016, weighted count (SE)**	**ED 2016, weighted count (SE)**	**Percent of cases**	**% of all deaths**	**RR (95% CI)**
**Cases**	10,525	155,610	139,160			
**Drugs per case**	1.40 (0.01)	1.21 (0.003)	1.46 (0.004)			
**4-Aminophenol derivative**	393	16,159 (273)	26,950 (342)	11.1%	3.7%	0.32 (0.28, 0.35)
**Anti-allergy/anti-emetic**	602	8,461 (207)	10,580 (224)	6.1%	7.7%	1.33 (1.24, 1.43)
**Anti-depressant**	1,615	20,696 (311)	32,445 (370)	15.5%	16.3%	1.10 (1.05, 1.16)
**Anti-diabetic**	87	1,603 (85)	4,400 (147)	1.7%	1.1%	0.69 (0.56, 0.82)
**Anti-epileptic**	471	8,942 (212)	12,145 (239)	8.0%	6.5%	0.84 (0.78, 0.90)
**Anti-Parkinson’s**	140	3,933 (136)	4,775 (153)	1.9%	1.2%	0.62 (0.52, 0.73)
**Salicylates**	100	3,397 (128)	6,085 (172)	2.2%	0.7%	0.30 (0.23, 0.38)
**Barbiturate**	150	555 (50)	1,865 (96)	0.4%	1.5%	3.69 (3.14, 4.25)
**Benzodiazepine**	1,245	39,026 (414)	58,700 (459)	19.6%	14.7%	0.74 (0.70, 0.78)
**Beta blockers**	180	1,192 (75)	2,955 (120)	1.4%	4.2%	1.25 (1.06, 1.43)
**Calcium channel blockers**	134	380 (42)	1,925 (80)	0.6%	7.4%	2.24 (1.87, 2.61)
**Cannabis**	26	1,903 (93)	1,640 (90)	1.2%	0.7%	0.22 (0.14, 0.30)
**Cocaine**	228	4,495 (144)	8,200 (198)	2.0%	4.5%	2.39 (2.18, 2.60)
**Hallucinogen**	2	576 (52)	350 (42)	0.3%	0.0%	0.06 (0.00, 0.16)
**Ibuprofen**	39	13,515 (248)	8,680 (204)	7.7%	0.3%	0.04 (0.03, 0.06)
**Muscle relaxants**	7	2,646 (113)	5,505 (164)	1.8%	2.1%	1.22 (1.06, 1.38)
**Opioid**	3,226	35,350 (381)	33,250 (374)	17.3%	47.8%	4.55 (4.38, 4.73)
**Other analgesics**	7	746 (62)	510 (50)	0.3%	0.0%	0.13 (0.03, 0.26)
**Other tranquilizer**	500	11,202 (233)	19,250 (295)	9.7%	6.2%	0.64 (0.59, 0.69)
**Psychostimulant**	218	6,138 (171)	7,040 (184)	3.9%	4.0%	1.05 (0.95, 1.16)
**Other named drug**	472	17,174 (268)	19,030 (287)	12.0%	4.5%	0.35 (0.32, 0.39)
**Unknown drug only**	881	16,949 (265)	5,715 (166)	7.7%	8.4%	1.13 (1.06, 1.21)

©2020 Miller TR et al. *JAMA Network Open*.
